# Supplementary material for: A fault-intrusion-tolerant system and deadline-aware algorithm for scheduling scientific workflow in the cloud
Source: PeerJ Comput Sci. 2021 Nov 2;7:e747. doi: 10.7717/peerj-cs.747 (PMC8576554; doi:10.7717/peerj-cs.747)
Supplement: Supplemental Information 6 [file peerj-cs-07-747-s006.zip › Myproject1.1/docs/org/cloudbus/cloudsim/distributions/package-summary.html]

org.cloudbus.cloudsim.distributions


JavaScript is disabled on your browser.


- Overview
- Package
- Class
- Use
- Tree
- Deprecated
- Index
- Help

- Prev Package
- Next Package

- Frames
- No Frames

- All Classes

# Package org.cloudbus.cloudsim.distributions

- Interface Summary

  | Interface | Description |
  | --- | --- |
  |  |  |
  | --- | --- |
  | ContinuousDistribution | Interface to be implemented by a random number generator. |
- Class Summary

  | Class | Description |
  | --- | --- |
  |  |  |
  | --- | --- |
  | ExponentialDistr | An exponential number generator. |
  | GammaDistr | The Class GammaDistr. |
  | LognormalDistr | The Class LognormalDistr. |
  | LomaxDistribution | The Class LomaxDistribution. |
  | ParetoDistr | The Class ParetoDistr. |
  | UniformDistr | A random number generator based on the Uniform distribution. |
  | WeibullDistr | The Class WeibullDistr. |
  | ZipfDistr | The Class ZipfDistr. |

- Overview
- Package
- Class
- Use
- Tree
- Deprecated
- Index
- Help

- Prev Package
- Next Package

- Frames
- No Frames

- All Classes
